# The Prognostic Value of Clinical and Pathological Response to Neoadjuvant Therapy in Metastatic Renal Cell Carcinoma Undergoing Cytoreductive Nephrectomy: A Systematic Review and Clinical Implications

**DOI:** 10.3390/cancers18111829

**Published:** 2026-06-02

**Authors:** Daria Chernysheva, Pedro Hernandez-Peñalver, Pablo Maroto, Joan Palou, Alberto Breda, Oscar Rodriguez-Faba

**Affiliations:** 1 Institut de Recerca, Hospital Sant Pau, 08041 Barcelona, Spain; 2 Uro-Oncology Department, Fundació Puigvert, 08025 Barcelona, Spain

**Keywords:** metastatic renal cell carcinoma, cytoreductive nephrectomy, immunotherapy, pathological reporting metrics, survival outcomes

## Abstract

Background: In the immunotherapy era, cytoreductive nephrectomy (CN) for metastatic renal cell carcinoma (mRCC) is increasingly performed after neoadjuvant immune checkpoint inhibitor (ICI)-based therapy. Examination of the nephrectomy specimen may capture the depth of treatment-induced tumor clearance more accurately than size-based radiological criteria alone. However, pathological reporting is highly heterogeneous across studies: residual viable tumor (RVT), necrosis, pT stage, and binary downstaging have all been used, limiting reproducible cross-study comparison. We aimed to characterize this heterogeneity, assess its implications for evidence synthesis, and propose a pragmatic framework for qualitative interpretation. Methods: PRISMA-compliant systematic review of studies reporting pathological response and oncological outcomes in mRCC patients undergoing CN after neoadjuvant systemic therapy (PROSPERO CRD420251154068). A qualitative synthesis was performed. A three-category Pathological Response Category (PRC) framework is proposed to harmonize heterogeneous metrics. Results: Seven retrospective studies (*n* = 408) were included. Pathological reporting metrics were inconsistent across all studies, preventing formal meta-analysis. Nevertheless, across cohorts reporting survival outcomes, deeper pathological response was directionally associated with more favorable oncologic outcomes. A discordance between radiological and pathological response was observed, including near-complete tumor clearance in patients classified as radiologically stable, reflecting the non-size-based mechanisms of ICI-induced tumor killing. Conclusions: The central finding of this review is not that pathological response predicts survival—which is expected—but that current pathological reporting in the mRCC surgical setting is too heterogeneous to quantify the frequency, depth, or prognostic significance of that response in a reproducible way. Prospective adoption of standardized pathological reporting protocols is the most critical next step for this field.

## Simple Summary

In metastatic renal cell carcinoma, cytoreductive nephrectomy following neoadjuvant systemic therapy is increasingly practiced, yet the pathological assessment of surgical specimens remains unstandardized. Across published series, pathological response is variably defined using residual viable tumor percentage, necrosis extent, pathological stage, or binary downstaging—metrics that are not biologically equivalent, particularly after immune checkpoint inhibitor therapy, where treatment effect manifests as fibrosis, granulomatous inflammation, and immune infiltration rather than simple necrosis. This systematic review maps the heterogeneity of current reporting practices across seven retrospective cohorts and proposes a pragmatic Pathological Response Category framework to harmonize cross-study comparison. Standardization of pathological reporting is identified as the critical prerequisite for future biomarker-driven trials and response-adapted treatment strategies in this setting.

## 1. Introduction

Renal cell carcinoma (RCC) accounts for approximately 90% of all renal tumors, with metastatic disease present at diagnosis in 15% of patients [1,2]. Cytoreductive nephrectomy (CN) was historically a cornerstone of metastatic RCC (mRCC) management, but its role has been substantially redefined after the CARMENA trial (2018) demonstrated non-inferiority of sunitinib alone versus upfront CN plus sunitinib [3], while the SURTIME trial supported a deferred approach to CN after systemic therapy [4].

The advent of immune checkpoint inhibitors (ICIs) has transformed first-line mRCC management, achieving superior response rates and survival greatly exceeding those observed with tyrosine kinase inhibitors (TKIs), as established in pivotal phase III trials such as CheckMate 214 (nivolumab plus ipilimumab) and KEYNOTE-426 (pembrolizumab plus axitinib) [5,6,7]. In this context, CN is increasingly reserved for carefully selected patients who demonstrate a response to neoadjuvant ICI [8,9]. Within this framework, the response assessment may have both prognostic and practical implications for surgical selection.

Current decision-making still relies largely on radiological response, most commonly according to RECIST [10]. However, ICI-induced tumor regression may be accompanied by fibrosis, hyalinization, immune-cell infiltration, granulomatous inflammation, and other treatment-related changes that reduce viable tumor burden without producing proportional dimensional shrinkage on cross-sectional imaging [11,12,13,14]. As a result, RECIST may underestimate the true depth of biological response, while pathological assessment may capture biologically meaningful response. Whether and to what extent pathological response carries prognostic significance in this setting, and how it relates to radiological response, remains incompletely defined.

A major obstacle is the heterogeneity of pathological reporting across published studies. Residual viable tumor percentage (RVT%), necrosis %, pT stage, and binary downstaging are related but not interchangeable endpoints, particularly in the ICI era. The value of pathological response as a prognostic and regulatory endpoint has been established in other solid tumor types. In breast cancer, pathological complete response (pCR) after neoadjuvant chemotherapy predicts event-free and overall survival and has been accepted by regulatory agencies as a surrogate endpoint supporting accelerated drug approval [15,16]. In resectable non-small-cell lung cancer, major pathological response (MPR, ≤10% residual viable tumor) after neoadjuvant chemoimmunotherapy predicts disease-free survival across pivotal trials, including CHECKMATE-816 [17,18]. In muscle-invasive bladder cancer undergoing radical cystectomy, ypT0N0 status is independently associated with improved long-term survival and informs decisions about adjuvant therapy [19,20]. These precedents demonstrate that tissue-level assessment of treatment effect in resected specimens can provide clinically relevant prognostic information beyond radiological staging and suggest that an analogous role is biologically plausible in mRCC, provided the methodological barriers to standardized pathological reporting in this disease are overcome. The present systematic review addresses the first step toward that goal: mapping the current landscape of pathological reporting in the mRCC surgical setting, characterizing the discordance between radiological and pathological response, and proposing a Pathological Response Category (PRC) framework to harmonize heterogeneous reporting metrics.

## 2. Materials and Methods

### 2.1. Study Design

This systematic review was conducted and reported in accordance with the Preferred Reporting Items for Systematic Reviews (PRISMA) guidelines and registered with PROSPERO (CRD420251154068). Given the substantial clinical and methodological heterogeneity—encompassing different treatment regimens, response definitions, pathological reporting standards, and survival endpoints—we performed a qualitative synthesis rather than a quantitative meta-analysis.

### 2.2. Search and Screening Strategy

A systematic literature search was conducted in PubMed, Web of Science/MEDLINE, Cochrane Library CENTRAL, and EMBASE from database inception to 1 June 2025. The search was restricted to English-language publications. The search strategy combined MeSH headings with free-text terms across four conceptual domains: metastatic renal cell carcinoma, neoadjuvant systemic therapy, pathological response, and survival outcomes. The full PubMed search string is provided in Supplementary Table S1; equivalent strategies were adapted for each database.

Search results were imported into the Rayyan software (Rayyan Systems Inc., Cambridge, MA, USA; accessed in 2025) for duplicate removal and screening. Two reviewers (D.C. and P.H.) independently screened all titles and abstracts; disagreements were resolved through discussion with a senior investigator (O.R.-F.).

**Figure 1:**
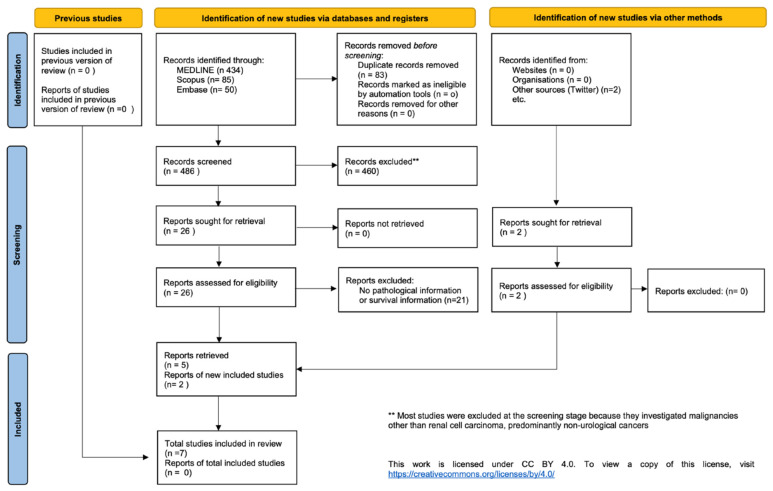
Results of the search and screening summarised in PRISMA chart.

Results of the search and screening are summarized in the PRISMA chart (Figure 1).

### 2.3. Eligibility Criteria

Studies were included if they met all of the following criteria:

- Population: Adults with metastatic RCC (any histologic subtype) diagnosed by imaging and/or histology.
- Intervention/exposure: Neoadjuvant systemic therapy prior to CN, including ICI, TKI, or combinations.
- Index measurements: At least one primary-tumor response metric used: radiologic (RECIST criteria) and/or pathologic (e.g., residual viable tumor [RVT %], necrosis, and/or ypT downstaging).
- Outcomes: Oncologic outcomes reported after CN, including progression-free survival (PFS), disease-free survival (DFS), or cancer-specific survival (CSS).

Single case reports, editorials, letters, review articles, and meeting abstracts were excluded at the initial screening. Only original studies that responded to the study question were included for a full-text evaluation and potential inclusion in the final synthesis. When the series combined upfront CN and deferred CN, data had to be separable for the deferred cohort to be eligible.

### 2.4. Data Extraction

The extracted data from full texts were collected in Excel (Microsoft Corporation, Redmond, CA, USA). We recorded study characteristics (year, country/centers, design, sample size); treatment details (regimen), primary-tumor response metrics: radiologic (size change or RECIST category), pathologic: RVT (%) and thresholds (e.g., pCR 0%, MPR ≤10%), necrosis, fibrosis, and ypT stage; outcomes after CN: PFS (DFS/CSS where available), OS, follow-up time, and events; hazard ratios with 95% CIs.

### 2.5. Risk of Bias and Study Quality

Given the predominance of observational designs, risk of bias was appraised qualitatively (Supplementary Figures S1 and S2). Judgments were structured using relevant domains from ROBINS-I and the Newcastle–Ottawa framework, with particular attention to patient selection, confounding, outcome ascertainment, and incomplete reporting. No composite numeric score was generated. Preprints were additionally flagged as non-peer-reviewed evidence.

### 2.6. Synthesis Approach and the PRC Classification Framework

Due to substantial clinical and methodological heterogeneity in treatment regimens, inconsistencies in pathological reporting standards, different survival endpoints, and mixed univariate/multivariable analyses, a quantitative meta-analysis was not performed. We therefore synthesized the evidence qualitatively, focusing on the direction of association between response depth and oncologic outcomes.

To facilitate qualitative comparison across heterogeneous studies, we developed a Pathological Response Category (PRC) framework. This conceptual framework places reported pathological metrics along a common spectrum of residual tumor viability rather than treating the underlying measurements as formally equivalent. The PRC system comprises three categories intended for interpretive, hypothesis-generating use only.

The three PRC categories are described in detail later in the manuscript (Section 3).

## 3. Results

### 3.1. Study Characteristics

The results of seven retrospective studies were included in this systematic review. Together, they included 408 patients with mRCC who underwent CN after neoadjuvant systemic therapy. Most series were single-institution cohorts, although multicenter and population-based data were also represented. Study periods ranged from the TKI era into the contemporary ICI era, and sample size varied markedly, from eight to 198 patients. Median follow-up ranged from 4.7 to 36 months, allowing only preliminary survival assessment in several cohorts.

Patient characteristics were consistent with advanced RCC presentations: median age at diagnosis ranged from 59 to 63 years, with a male predominance (more than 65% of patients). Histology was dominantly clear cell renal cell carcinoma (ccRCC); 24 (5.9%) patients were non-ccRCC. Clinical staging at the baseline reflected synchronous metastatic disease in 80–90% of patients, with primary tumor stages predominantly cT3. Distant metastases most commonly reported were located in the lungs, bones, or non-regional lymph nodes. The International Metastatic Renal Cell Carcinoma Database Consortium (IMDC) risk stratification, reported in five studies, classified the majority as intermediate risk (not less than 60% of patients in every study) and poor risk, reflecting real-world treatment patterns in advanced RCC. Detailed study characteristics and PRC tier assignability are presented in Table 1.

### 3.2. Neoadjuvant Treatment Regimens

Neoadjuvant regimens were heterogeneous and reflected the evolution of systemic therapy in mRCC. In the pooled cohort, 60% (*n* = 244) received ICI monotherapy or combinations, including the following: PD-1/PD-L1 inhibitors (nivolumab or pembrolizumab alone in 25 cases (10.2%, 25/244), CTLA-4 + PD-1 combination (ipilimumab + nivolumab in 38.9%, (95/244) of patients), and ICI + vascular endothelial growth factor (VEGF) combinations (e.g., pembrolizumab + axitinib, nivolumab + cabozantinib—in 11.5% (28/244)). Earlier TKI-based approaches (e.g., sunitinib, pazopanib, bevacizumab) were reported only in the study of Pieretti et al. (*n* = 164, 40%) [22]. The median interval from neoadjuvant therapy initiation to CN varied from 6 to 22.5 months.

### 3.3. Radiologic Response Assessment

All studies employed Radiological assessment of treatment response prior to CN, though evaluation methods, criteria, and timing differed substantially. RECIST v1.1 criteria were formally applied and categorical results reported in three studies [24,26,27]: partial response (≥30% size reduction) was documented in 45 patients, complete radiological response in one patient, stable disease in 29 patients, and progressive disease in five patients across these three cohorts combined.

The remaining studies used alternative radiological metrics. Hakimi et al. reported median change in primary tumor size and changes in RENAL nephrometry score [25], while Pieretti et al. used a binary threshold of ≥10% tumor shrinkage in the primary and/or metastatic lesions to define radiological response [22].

Table 1 summarizes the radiological response data across studies alongside corresponding pathological findings. Because radiological response was reported using non-uniform metrics, direct cross-study comparison of radiological response rates was not possible.

### 3.4. The PRC Classification Framework

To facilitate cross-study comparison despite heterogeneous pathological reporting, we propose the Pathological Response Category (PRC) classification. It is anchored to the residual viable tumor (RVT%) while recognizing that the observed non-viable component may reflect different biological processes. The PRC framework applies only to pathological metrics derived from the resected specimen: RVT%, necrosis%, ypT stage, and binary pathological downstaging. Radiological metrics (RECIST categories, tumor size change) are intentionally excluded as they describe preoperative tumor dimensions rather than tissue-level viability. The PRC framework should therefore be regarded as a pragmatic interpretive model, not as a validated grading system.

All studies are assignable to at least one PRC: Pieretti 2021 via pT stage [22]; Singla 2019 via pT stage and necrosis presence [21]; Panian 2024 and Hakimi 2023 via pathologic downstaging and pT0 rates [24,25]; Khandwala 2025 via explicit RVT% [23]; Amaral 2025 and Jain 2025 via necrosis% [26,27]. However, the degree of alignment varied across studies, and in some cohorts, the available metrics permitted only broad categorization.

The PRC framework does not assume arithmetic equivalence between necrosis and RVT. In TKI-treated tumors, non-viable tissue is predominantly necrotic, and necrosis% closely approximates the complement of RVT%. In ICI-treated tumors, however, the non-viable compartment treated tumors may exhibit fibrosis, hyalinization, granulomatous inflammation, vasculitic change, and immune-cell infiltration in addition to or instead of necrosis. For this reason, the PRC categories should be interpreted as reflecting the overall depth of pathological clearance rather than a single histological mechanism. The three conceptual PRC categories are summarized in Table 2.

### 3.5. Pathologic Response

Pathological evaluation of CN specimens provided the most granular information across studies, but it was also the least standardized domain of reporting. Metrics included RVT, major pathological response (MPR, defined as <10% RVT or complete/near-complete necrosis [23]), pT stage, and qualitative features (proportion of necrosis, vasculitis, or granulomas). Some papers were mainly focused on viable tumor burden [23], necrosis [26,27], or pT stage [21,22,24,25]. However, some authors additionally used a binary evaluation system as “pathological downstaging” [24,25].

RVT was quantified only by Khandwala et al., describing the results with several thresholds: <10% RVT (=MPR)—15 (26%), 10–50% RVT—16 (28%) of patients, and 50% RVT for 27 (47%) of patients [23].

Pathological complete response (pT0 or 0% RVT) was uncommon (7.1% (29/408) of the total cohort), but reported across multiple studies. The degree of necrosis, where reported, ranged from 5% to 100%, with Amaral et al. reporting necrosis in 96% of patients and >95% necrosis in 42% [27]. Final pT stage was documented in four studies (*n* = 316), with pT3a–c representing 50.1%,s demonstrating that viable tumor burden frequently persists even in surgically selected patients [21,22,24,25].

### 3.6. Survival Outcomes and PRC-Mapped Associations

Survival outcomes grouped by PRC-comparable response depth and outcome type are summarized in Table 3. Although response definitions, analytical models, and endpoints differed across studies, the overall direction of association was consistent: deeper pathological response tended to be associated with more favorable progression-free, cancer-specific, or overall survival.

The most statistically compelling data derive from Khandwala et al. (2025), the ICI-focused study, in which MPR was associated with improved PFS (HR = 0.05 (95% CI 0.01–0.41, *p* = 0.005) and OS (HR = 0.07 (95% CI 0.01–0.88, *p* = 0.04) [23]. These estimates should be interpreted cautiously because of the small number of MPR events (*n* = 13), wide confidence intervals; the direction of effect is, however, clinically relevant. Pieretti et al. (2021), using a TKI-dominant cohort with a longer follow-up period, reported adjusted HRs of 0.44–0.48 for OS and CSS in patients achieving ≥10% tumor shrinkage [22]. Hakimi et al. (2023) observed the association between pathological downstaging and PFS (HR = 5.15), while continuous radiological change (per cm reduction) contributed an HR = 1.20, suggesting that pathological response could carry a stronger prognostic signal than radiological metrics alone in this dataset [25].

## 4. Discussion

The primary contribution of this review is methodological: We demonstrate that pathological reporting after neoadjuvant therapy before CN in mRCC is too heterogeneous to support cross-study comparison or formal meta-analysis. RVT, necrosis, pT stage, and binary downstaging are all being used, but they do not describe the same biological feature with the same level of granularity, particularly after ICI-based therapy.

RECIST was developed in the cytotoxic chemotherapy era, where tumor cell death is predominantly reflected by volumetric shrinkage. ICI-based therapies induce immune-mediated clearance that may eliminate viable tumors without proportional size reduction on CT [13,28]. Intratumoral heterogeneity further complicates this: Immune infiltration and fibrotic replacement are non-uniform, meaning that dimensional measurements of the outer tumor boundary may not reflect the viable cell burden within [13,14,29]. iRECIST addresses pseudoprogression but does not account for the more common pattern of size-stable but biologically responsive tumors [30].

Radiological and pathological responses are imperfectly and inconsistently correlated in this setting and this review provides direct evidence of the discordance. Amaral et al. documented necrosis in 96% of specimens and ≥95% necrosis in 42%, yet a substantial proportion of these patients had stable disease by RECIST pre-operatively [26]. Other studies report that RECIST underestimates ICI benefit in up to 15% of patients [11,31]. Only one study provided a Spearman correlation of 0.51 between change in primary tumor diameter and residual viable tumor percentage, indicating that radiological shrinkage explains less than 30% of the variance in pathological response [23]. Patients with radiographically stable disease frequently demonstrated substantial necrosis on final pathology, while some with apparent partial radiographic response retained high residual viable tumor burden. The potential clinical implication is, in the deferred CN setting, where radiological response currently serves as the primary criterion for surgical candidacy, a meaningful proportion of true biological responders may be excluded from surgery, while patients with high residual viable tumor burden despite apparent radiological response may proceed to an operation of limited oncological benefit. However, this hypothesis requires prospective testing in non-operated populations.

The biological interpretation of pathology also differs between treatment classes. In the TKI-era series, treatment effect was often expressed predominantly as necrosis, making necrosis a rough surrogate for reduced viability. In ICI-treated tumors, however, the remnant tissue appears to be more complex. Jain et al. report immune-related vasculitis and non-necrotizing granulomas in post-ICI CN specimens—features absent from TKI-era material and not captured by any existing RCC reporting system [27]. Reporting frameworks limited to necrosis% or RVT%, therefore, may fail to capture biologically meaningful treatment responses in ICI-treated patients. The spatial patterns of tumor regression, such as centripetal versus non-centripetal clearance, and unifocal versus multifocal or scattered regression, have been formally characterized in other tumor types following neoadjuvant therapy, but have not yet been systematically applied to RCC [32,33,34]. Whether such regression topography carries independent prognostic information in this disease remains unknown and represents a potentially valuable direction for future standardized reporting protocols.

Pathological response should accordingly be viewed as a promising but still immature endpoint. It may ultimately prove useful fouf or postoperative risk stratification, correlative biomarker work, and trial design, particularly if linked to preoperative predictors such as IMDC risk, radiological kinetics, circulating tumor DNA dynamics, or regimen class. None of these translational questions can be addressed robustly, however, without standardized pathology reporting.

Beyond its role as a possible prognostic correlate, pathological response could serve an additional function in the postoperative management of mRCC. In the context of deferred CN, where surgery is performed after a period of systemic therapy, the depth of pathological response in the resected specimen provides information that was not available at the time of surgical candidacy assessment. Patients with incomplete pathological clearance represent a population in whom intensification or modification of subsequent systemic therapy may be warranted, and in whom closer surveillance would be clinically justified. Conversely, patients achieving major or complete pathological response may represent a subgroup in whom de-escalation of maintenance therapy could be explored. This kind of pathological response-adapted treatment strategy is already operational in other solid tumors: In early-stage HER2-positive breast cancer, residual disease after neoadjuvant therapy triggers escalation to adjuvant trastuzumab emtansine [35]. Whether a similar framework is feasible and beneficial in mRCC remains entirely unexplored, but it represents a scientifically coherent and clinically important question that standardized pathological reporting would directly enable. At a minimum, systematic documentation of pathological response depth would allow treating oncologists to incorporate specimen findings into individualized postoperative discussions—a practice that is currently impossible due to the absence of a shared reference standard.

Several ongoing and recently initiated prospective studies provide the infrastructure within which standardized pathological assessment could be implemented. The PROBE trial (NCT03456804) and NORDIC-SUN trial are evaluating deferred CN in patients receiving first-line ICI-based therapy; neither currently mandates a standardized pathological reporting protocol, but both collect nephrectomy specimen data that could be retrospectively harmonized using a PRC-like framework [36]. The INKCR guidelines provide an immediately actionable reference standard: They recommend quantification of residual viable tumor in 10% intervals, reporting of the greatest linear extent of viable tumor, and documentation of non-viable compartment composition (necrosis, fibrosis, immune infiltrate) [28]. Prospective adoption of these guidelines by future trial investigators would transform the surgical cohort from a descriptive dataset into a repository capable of supporting meaningful pathological response analysis.

Interpretation of the directional associations between pathological features and survival outcomes reported here is further constrained by confounders that the available evidence cannot adequately address. IMDC risk group, reported in only five of seven studies and predominantly intermediate across all cohorts, metastatic burden, histological subtype, and the interval from therapy initiation to surgery varied substantially across series but were inconsistently captured, precluding adjusted analysis. The heterogeneity of systemic regimens (from TKI monotherapy to IO-IO and IO-TKI combinations) independently influences both the depth and biological character of pathological response, rendering cross-study survival comparisons inherently confounded. The incorporation of biomarker studies in ongoing and future clinical trials represents the logical next step toward response-adapted treatment strategies in this disease.

This review has several limitations. All included studies were retrospective and restricted to patients selected for surgery, introducing substantial selection bias. A fundamental consequence of this design is that patients who do not respond to neoadjuvant therapy are frequently not offered cytoreductive nephrectomy; their specimens are therefore never available for pathological assessment. This creates an inherent ascertainment bias that prevents any estimation of the sensitivity or specificity of pathological response as a predictive biomarker in the broader mRCC population. The findings of this review are therefore applicable only to the operated, pre-selected cohort and cannot be extrapolated to guide surgical decision-making in patients who have not yet undergone surgery. Response definitions were heterogeneous, which precluded meta-analysis and limited quantitative inference. Follow-up was generally shorter in contemporary ICI-era cohorts than in older TKI-era series, and important clinical variables such as IMDC risk were inconsistently reported. Finally, the PRC framework proposed here is conceptual and requires prospective validation. Future progress will depend on the systematic implementation of standardized nephrectomy-specimen reporting, ideally aligned with recently proposed kidney cancer pathology recommendations [28].

## 5. Conclusions

The directional association between deeper pathological response and better oncological outcomes is consistent across all included cohorts and provides biological plausibility for pathological response as a prognostic biomarker. However, this association cannot be quantified with precision from the available data, and the discordance observed between radiological and pathological response raises unresolved questions about current patient selection criteria for CN. These questions cannot be answered without prospective and standardized pathological data collection.

The Pathological Response Category framework proposed here represents a hypothesis-generating attempt to harmonize these heterogeneous metrics; it requires prospective validation before clinical application. Adoption of the International Neoadjuvant Kidney Cancer Consortium [28] reporting guidelines and incorporation of pathological endpoints into ongoing prospective trials (PROBE, NORDIC-SUN) are the most actionable next steps.

## Figures and Tables

**Table 1:** Characteristics of included studies: design, sample size, treatment regimens, and pathological response metrics.

Author (Year, Country)	Journal	Study Design and Period	*n*	Follow up, Month	Neoadj. Regimen	Radiol. Response	Pathol. Response	PRC
Singla (2019, USA) [21]	Urologic Oncology	Retrospective single center, 2016–2018	10	6.7 (3.7–8.2)	ICI (PD-1/PD-L1)	RECISTv1	pT/pN stage	Complete/Major/Incomplete (pT stage)
Pieretti (2021, USA) [22]	Urologic Oncology	Retrospective single center, 2005–2019	198	33.7 (16.2–60.1)	TKI (sunitinib, pazopanib); some ICI	RECISTv1	Tumor shrinkage > 10%, pT stage	Complete/Major/Incomplete (pT stage; pT0 not reported separately; primary survival analysis by radiologic shrinkage)
Khandwala (2025, USA) [23]	medRxiv (preprint)	Retrospective single center, 2015–2024	60	29 (22–44)	ICI-based (ICI–ICI and ICI–TKI)	RECISTv1	RVT%; MPR =<10% RVT, pCR	Complete/Major/Incomplete (RVT%)
Panian (2024, USA) [24]	The Oncologist	Retrospective multi center, 2016–2021	52	Median 25.3	ICI-based (PD-1/CTLA-4 and ICI–TKI)	RECISTv1	Pathologic downstaging (binary)	Major/Incomplete (downstaging; pT0 = 13%)
Hakimi (2023, USA) [25]	Clinical Genito urinary Cancer	Retrospective multi center, 2016–2021	56	22.5 (22.2–32.7)	ICI-based (predominantly ICI–TKI)	change in tumor size (cm, %), change in RENAL score	pT stage	Major/Partial (pT downstaging; 8 pT0 cases)
Amaral (2025, USA) [26]	The Oncologist	Retrospective single center, 2018–2024	24	25.8 (4.6–92.6)	ICI-based (ICI–TKI and ICI–ICI)	RECISTv1	pT stage, necrosis %	Complete/Major/Incomplete (necrosis%; pT0 = 21%)
Jain (2025, USA) [27]	Pathologica	Retrospective single center, 2019–2024	8	2–20	ICI-based	RECISTv1	necrosis % (range 5–90%)	Incomplete response (necrosis%; no threshold)

pCR—pathologic complete response; RVT—residual viable tissue; MPR—major pathologic response; ICI—immune checkpoint inhibitors; TKI—Tyrosine kinase inhibitors.

**Table 2:** Proposed Pathological Response Category (PRC) framework: definitions, biological rationale, and correspondence to existing reporting metrics.

PRC	Definition	Operative Metrics	Studies Applicable
Complete Response	No residual viable malignant cells. The lesion is replaced entirely by necrosis, fibrosis, hyalinization, or immune infiltrate. Corresponds to pT0 or pCR.	RVT = 0%; necrosis = 100%; pT0; pCR	Khandwala 2025 (pCR subgroup) Amaral 2025 (pT0, *n* = 5; 21%) Singla 2019 (pT0 cases) [21,23,26]
Major Response	Near-complete tumor clearance. The lesion retains minimal viable tumor. Non-viable compartment may include necrosis, fibrosis, granulomatous inflammation, or hyalinization.	RVT ≤ 10% (MPR); necrosis ≥ 90–95%; near-complete pT downstaging	Khandwala 2025 (MPR, *n* = 15; 26%) Amaral 2025 (necrosis > 95%, *n* = 10; 42%) Jain 2025 (high necrosis subgroup) [23,26,27]
Incomplete Response	Incomplete tumor clearance. Viable tumor remains a major component of the specimen. Treatment response changes (necrosis, fibrosis) are present but do not dominate. This group may include both moderate responders and true non-responders due to heterogeneous thresholds.	RVT > 10%; necrosis < 90%; pathologic downstaging without pT0/MPR; or pT3–4 without dominant regression features	Khandwala 2025 (RVT 10–50%, *n* = 16; and RVT > 50%, *n* = 27) Panian 2024 (non-pT0 cases) Hakimi 2023 (all non-pT0) Pieretti 2021 (non-shrinkage and shrinkage groups) Singla 2019 (non-pT0 cases) [21,22,23,24,25]

pCR—pathologic complete response; RVT—residual viable tissue; MPR—major pathologic response.

**Table 3:** Survival outcomes stratified by pathological response: hazard ratios and 95% confidence intervals across included studies.

Study (Year)	PRC Comparison	Events (*n*)	Outcome	HR (95% CI)	Analysis Type	Regimen Class
Overall Survival						
Pieretti 2021 [22]	Major Response vs. Incomplete Response (primary + metastatic shrinkage ≥ 10%)	56	OS	0.44 (0.29–0.67)	Multivariable	TKI
Pieretti 2021 [22]	Major Response vs. Incomplete Response (primary tumor shrinkage ≥ 10%)	62	CSS	0.48 (0.32−0.73)	Multivariable	TKI
Khandwala 2025 [23]	Complete or Major Response (MPR, RVT < 10%) vs. Incomplete Response	13	OS	0.07 (0.01–0.88) *	Multivariable	ICI-based
Progression-Free Survival						
Khandwala 2025 [23]	Complete or Major Response (MPR) vs. Incomplete Response	13	PFS	0.05 (0.01–0.41) *	Multivariable	ICI-based
Hakimi 2023 [25]	Incomplete Response (pathologic downstaging) vs. Major Response	25	PFS	5.15 (1.29–20.6)	Multivariable	ICI-based
Pieretti 2021 [22]	Major Response vs. Incomplete Response (primary + metastatic shrinkage)	56	CSS	0.44 (0.29–0.67)	Multivariable	TKI

PFS—progression-free survival; CSS—cancer-specific survival; ICI—immune checkpoint inhibitors; TKI—Tyrosine kinase inhibitors. * Wide CI reflects small event number (*n* = 13 MPR cases); interpret with caution.

## Data Availability

No new data were created or analyzed in this study.

## Supplementary Materials

The following supporting information can be downloaded at: https://www.mdpi.com/article/10.3390/cancers18111829/s1, Table S1. Search strategy. Figure S1. Risk of bias according to ROBINS-I (v2); Figure S2. Risk of bias according to the Newcastle–Ottawa framework.

## Abbreviations

CI	Confidence interval
CN	Cytoreductive nephrectomy
ccRCC	Clear cell renal cell carcinoma
cT	Clinical T stage
cT3	Clinical T stage 3
CSS	Cancer-specific survival
CTLA-4	Cytotoxic T-lymphocyte-associated protein 4
DFS	Disease-free survival
HR	Hazard ratio
ICI	Immune checkpoint inhibitor(s)
IMDC	International Metastatic Renal Cell Carcinoma Database Consortium
mRCC	Metastatic renal cell carcinoma
MPR	Major pathologic response
OS	Overall survival
pCR	Pathologic complete response
PD-1	Programd cell death protein 1
PD-L1	Programd death-ligand 1
PFS	Progression-free survival
pN	Pathological N stage
pT	Pathological T stage
PRISMA	Preferred Reporting Items for Systematic Reviews and Meta-Analyses
RCC	Renal cell carcinoma
RECIST	Response Evaluation Criteria in Solid Tumors
RENAL	RENAL nephrometry score
RVT	Residual viable tumor
TKI	Tyrosine kinase inhibitor(s)
TMT	Targeted molecular therapy/therapies
VEGF	Vascular endothelial growth factor
ypT	Post-treatment pathological T stage
