# Supplementary material for: The Prognostic Value of Clinical and Pathological Response to Neoadjuvant Therapy in Metastatic Renal Cell Carcinoma Undergoing Cytoreductive Nephrectomy: A Systematic Review and Clinical Implications
Source: Cancers (Basel). 2026 Jun 2;18(11):1829. doi: 10.3390/cancers18111829 (PMC13257015; doi:10.3390/cancers18111829)
Supplement: Supplementary file 1 [file cancers-18-01829-s001.zip › cancers-4300313-supplementary.pdf]

SUPPLEMENTARY MATERIALS

SUPPLEMENTARY TABLE

Suplementary Table S1. Search strategy

Pubmed	((("Carcinoma, Renal Cell"[Mesh] OR "Kidney Neoplasms"[Mesh]) AND ("Neoplasm Metastasis"[Mesh] OR metastatic[tiab] OR metastasis[tiab]))<br>OR "renal cell carcinoma"[tiab] OR RCC[tiab]) AND ("Neoadjuvant Therapy"[Mesh] OR"neoadjuvant"[tiab] OR "preoperative"[tiab] OR "induction therapy"[tiab] OR "pre-surgical"[tiab] OR "presurgical"[tiab]) AND ("Pathologic Stage"[Mesh] OR "pT stage"[tiab] OR "pathological stage"[tiab] OR "pathologic T stage"[tiab] OR "ypT"[tiab] OR "pathologic response"[tiab] OR "histological response"[tiab] OR "histopathologic response"[tiab])<br>AND ("Survival Analysis"[Mesh] OR "Progression-Free Survival"[Mesh] OR "Overall Survival"[Mesh] OR survival[tiab] OR "progression free"[tiab] OR PFS[tiab] OR OS[tiab] OR "outcome"[tiab] OR "outcomes"[tiab] OR "prognosis"[tiab] OR "prognostic"[tiab])
Medline	1. exp Carcinoma, Renal Cell/<br>2. exp Kidney Neoplasms/<br>3. renal cell carcinoma.tw.<br>4. RCC.tw.<br>5. ccRCC.tw.<br>6. 1 OR 2 OR 3 OR 4 OR 5<br>7. exp Neoplasm Metastasis/<br>8. metastatic.tw.<br>9. metastasis.tw.<br>10. advanced.tw.<br>11. mRCC.tw.<br>12. 7 OR 8 OR 9 OR 10 OR 11<br>13. exp Neoadjuvant Therapy/<br>14. neoadjuvant.tw.<br>15. preoperative.tw.<br>16. presurgical.tw.<br>17. pre-surgical.tw.<br>18. induction therapy.tw.<br>19. 13 OR 14 OR 15 OR 16 OR 17 OR 18<br><br>20. pathologic response.tw.<br>21. pathological response.tw.

	22. major pathologic response.tw.<br>23. major pathological response.tw.<br>24. complete pathologic response.tw.<br>25. complete pathological response.tw.<br>26. pathologic complete response.tw.<br>27. pathological complete response.tw.<br>28. residual viable tumor.tw.<br>29. residual viable tumour.tw.<br>30. necrosis.tw.<br>31. downstaging.tw.<br>32. ypT.tw.<br>33. tumor regression.tw.<br>34. tumour regression.tw.<br>35. histopathologic response.tw.<br>36. treatment response.tw.<br>37. 20 OR 21 OR 22 OR 23 OR 24 OR 25 OR 26 OR 27 OR 28 OR 29 OR 30 OR 31 OR 32 OR 33 OR 34 OR 35 OR 36<br><br>38. exp Survival Analysis/<br>39. exp Progression-Free Survival/<br>40. exp Overall Survival/<br>41. survival.tw.<br>42. progression free.tw.<br>43. PFS.tw.<br>44. OS.tw.<br>45. outcome*.tw.<br>46. prognosis.tw.<br>47. prognostic.tw.<br>48. 38 OR 39 OR 40 OR 41 OR 42 OR 43 OR 44 OR 45 OR 46 OR 47<br><br>49. 6 AND 12 AND 19 AND 37 AND 48
EMBASE	1. 'renal cell carcinoma'/exp<br>2. 'kidney tumor'/exp<br>3. 'renal cell carcinoma':ti,ab<br>4. RCC:ti,ab<br>5. ccRCC:ti,ab<br>6. 1 OR 2 OR 3 OR 4 OR 5<br>7. 'metastasis'/exp<br>8. metastatic:ti,ab<br>9. metastasis:ti,ab<br>10. advanced:ti,ab<br>11. mRCC:ti,ab<br>12. 7 OR 8 OR 9 OR 10 OR 11<br><br>13. 'neoadjuvant therapy'/exp<br>14. neoadjuvant:ti,ab<br>15. preoperative:ti,ab<br>16. presurgical:ti,ab

	17. pre-surgical:ti,ab<br>18. 'induction therapy':ti,ab<br>19. 13 OR 14 OR 15 OR 16 OR 17 OR 18<br><br>20. 'pathologic response':ti,ab<br>21. 'pathological response':ti,ab<br>22. 'major pathologic response':ti,ab<br>23. 'major pathological response':ti,ab<br>24. 'complete pathologic response':ti,ab<br>25. 'complete pathological response':ti,ab<br>26. 'pathologic complete response':ti,ab<br>27. 'pathological complete response':ti,ab<br>28. 'residual viable tumor':ti,ab<br>29. 'residual viable tumour':ti,ab<br>30. necrosis:ti,ab<br>31. downstaging:ti,ab<br>32. ypT:ti,ab<br>33. 'tumor regression':ti,ab<br>34. 'tumour regression':ti,ab<br>35. 'histopathologic response':ti,ab<br>36. 'treatment response':ti,ab<br>37. 20 OR 21 OR 22 OR 23 OR 24 OR 25 OR 26 OR 27 OR 28 OR 29 OR 30 OR 31 OR 32 OR 33 OR 34 OR 35 OR 36<br><br>38. 'survival analysis'/exp<br>39. 'progression free survival'/exp<br>40. 'overall survival'/exp<br>41. survival:ti,ab<br>42. 'progression free':ti,ab<br>43. PFS:ti,ab<br>44. OS:ti,ab<br>45. outcome*:ti,ab<br>46. prognosis:ti,ab<br>47. prognostic:ti,ab<br>48. 38 OR 39 OR 40 OR 41 OR 42 OR 43 OR 44 OR 45 OR 46 OR 47<br><br>49. 6 AND 12 AND 19 AND 37 AND 48
Cochrane Library CENTRAL	(("renal cell carcinoma" OR RCC OR ccRCC OR "kidney neoplasm*" OR "kidney cancer")<br>AND (metastatic OR metastasis OR advanced OR mRCC)<br>AND (neoadjuvant OR preoperative OR presurgical OR "pre-surgical" OR "induction therapy")<br>AND ("pathologic response"<br>OR "pathological response"<br>OR "major pathologic response"<br>OR "major pathological response"

	OR "complete pathologic response"<br>OR "complete pathological response"<br>OR "pathologic complete response"<br>OR "pathological complete response"<br>OR "residual viable tumor"<br>OR "residual viable tumour"<br>OR necrosis<br>OR downstaging<br>OR ypT<br>OR "tumor regression"<br>OR "tumour regression"<br>OR "histopathologic response"<br>OR "treatment response")<br>AND (survival<br>OR prognosis<br>OR prognostic<br>OR outcome*<br>OR "progression free"<br>OR PFS<br>OR OS)<br>)

SUPPLEMENTARY FIGURES

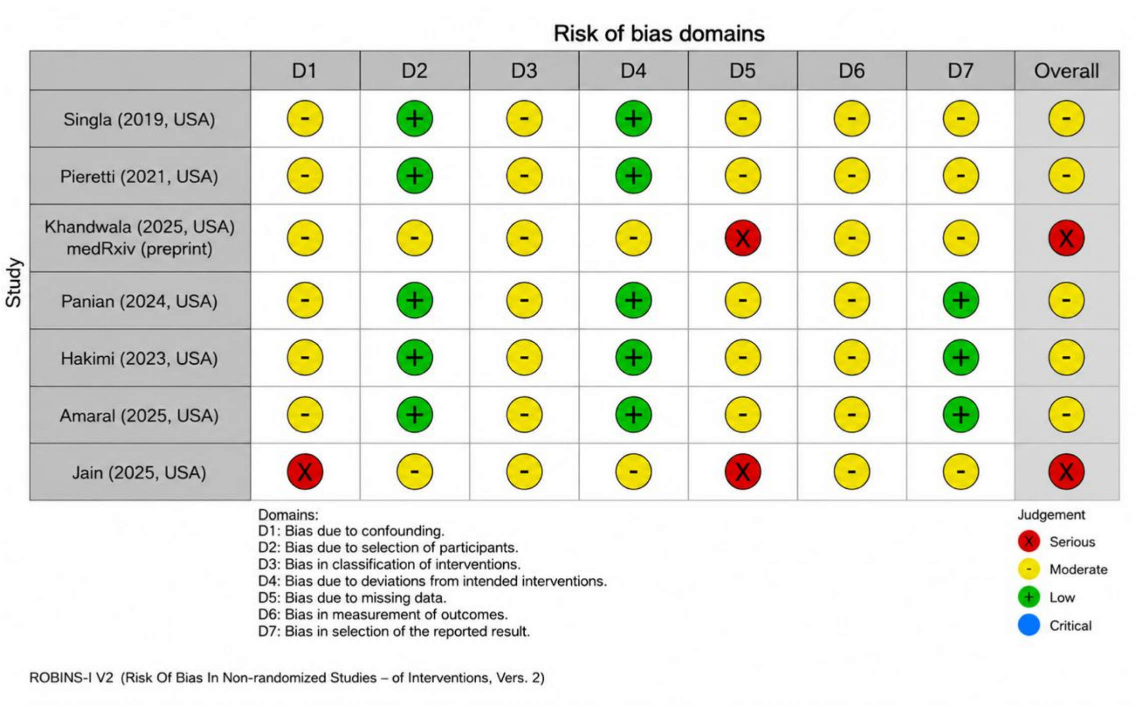

Figure S1. Risk of bias according to ROBINS-I (v2).

Newcastle–Ottawa Scale (NOS)

Study	Selection (4 stars)				Comparability (2 stars)		Outcome (3 stars)			Overall Quality
	S1 Representativeness of the exposed cohort	S2 Selection of the non-exposed cohort	S3 Ascertainment of exposure	S4 Demonstration that outcome of interest was not present at start of study	C1 Comparability of cohorts on the basis of the design or analysis	C2 Additional comparability (study controls for another factor)	O1 Assessment of outcome	O2 Was follow-up long enough for outcomes to occur	O3 Adequacy of follow-up of cohorts	
Singla (2019, USA)	+	-	+	+	+	-	+	+	-	-
Pieretti (2021, USA)	+	-	+	+	+	-	-	+	-	-
Khandwala (2025, USA) medRxiv (preprint)	-	-	+	+	-	x	+	-	-	x
Panian (2024, USA)	+	+	+	+	+	-	+	+	+	+
Hakimi (2023, USA)	+	+	+	+	+	-	+	+	+	+
Amaral (2025, USA)	-	-	+	+	+	-	+	-	-	-
Jain (2025, USA)	x	x	+	-	x	x	-	-	-	x

Selection (maximum 4 stars)
S1. Representativeness of the exposed cohort
S2. Selection of the non-exposed cohort
S3. Ascertainment of exposure
S4. Demonstration that outcome of interest was not present at start of study

Comparability (maximum 2 stars)
C1. Comparability of cohorts on the basis of the design or analysis
C2. Additional comparability (study controls for another factor)

Outcome (maximum 3 stars)
O1. Assessment of outcome
O2. Was follow-up long enough for outcomes to occur
O3. Adequacy of follow-up of cohorts

Overall Quality
+ High (7–9 stars)
- Moderate (4–6 stars)
x Low (0–3 stars)

Note: NOS = Newcastle–Ottawa Scale for cohort studies.

Figure S2. Risk of bias according to the Newcastle–Ottawa framework.
